# The association between institutional delivery and neonatal mortality based on the quality of maternal and newborn health system in India

**DOI:** 10.1038/s41598-022-10214-y

**Published:** 2022-04-13

**Authors:** Hwa-Young Lee, Hannah H. Leslie, Juhwan Oh, Rockli Kim, Alok Kumar, S. V. Subramanian, Margaret E. Kruk

**Affiliations:** 1 Harvard TH Chan School of Public Health, Boston, MA USA; 2 Institute of Convergence Science (ICONS), Convergence Science Academy, Yonsei University, Seoul, Korea; 3 Division of Prevention Science, University of California, San Francisco, San Francisco, CA USA; 4 Department of Medicine, Seoul National University College of Medicine, Seoul, Korea; 5 Division of Health Policy and Management, College of Health Sciences, Korea University, Seoul, Korea; 6 Harvard Center for Population and Development Studies, Cambridge, MA USA; 7 Interdisciplinary Program in Precision Public Health, Department of Public Health Sciences, Graduate School of Korea University, Seoul, Korea; 8 Health and Family Welfare, Government of Uttar Pradesh, Lucknow, Uttar Pradesh India

**Keywords:** Health care, Health policy, Health services, Paediatrics, Public health

## Abstract

Over 600,000 newborns in India died in their first month of life in 2017 despite large increases in access to maternal health services. We assess whether maternal and newborn health system quality in India is adequate for institutional delivery to reduce neonatal mortality. We identified recent births from the cross-sectional 2015–2016 National Family Health Survey and used reported content of antenatal care and immediate postpartum care averaged at the district level to characterize health system quality for maternity and newborn services. We used random effect logistic models to assess the relationship between institutional delivery and neonatal (death within the first 28 days of life) and early neonatal (death within 7 days of live births) mortality by quintile of district maternal and newborn health system quality. Three quarters of 191,963 births were in health facilities; 2% of newborns died within 28 days. District-level quality scores ranged from 40 to 90% of expected interventions. Institutional delivery was not protective against newborn mortality in the districts with poorest health system quality, but was associated with decreased mortality in districts with higher quality. Predicted neonatal mortality in the highest quintile of quality would be 0.018 (95% CI 0.010, 0.026) for home delivery and 0.010 (0.007, 0.013) for institutional delivery. Measurement of quality is limited by lack of data on quality of acute and referral care. Institutional delivery is associated with meaningful survival gains where quality of maternity services is higher. Addressing health system quality is an essential element of achieving the promise of increased access to maternal health services.

## Introduction

South Asia accounts for 38% of the estimated 2.5 million neonates dying in the first month of life globally, with over 600,000 neonatal deaths in India alone in 2017 (23.7 per 1000 live births) and nearly the same number of stillbirths ^1–4^. The main causes of neonatal death have not changed in India from 2000 to 2015 ^5^, and the majority can be averted through good quality health care before but especially during and after delivery ^6^. Progress has been slower in reducing intrapartum and neonatal deaths than other preventable deaths in India. Over 10% more neonates die than would be expected based on the under-5 mortality ratio, and substantial acceleration in survival will be required if the country is to achieve the Sustainable Development Goal target of 12 neonatal deaths per 1000 live births by 2030 compared to the 2017 rate of 24 (90% CI 21.3, 26.8) ^1,5^. Mortality has declined more quickly in urban areas and richer states, widening inequities for poorer and more rural areas of the country ^5,7,8^.

Beginning in the early 2000s, the Indian government implemented the National Rural Health Mission (2004), the Janani Suraksha Yojana (JSY) cash transfer program (2005), the Accredited Social Health Activist community health program (2006), and the Rashtriya SwasthyaBima Yojna insurance scheme (2008) in an effort to strengthen health systems in priority states, incentivize maternal health service use, expand community health services, and improve financial protection for inpatient services ^9^. Nationally representative studies support the role of these programs in increasing uptake of health services, particularly institutional delivery (39% in 2005 and 78.9% in 2015) ^10–12^, with some progress in narrowing wealth-based inequities in health service use ^13–16^. Whether this increased utilization translated into lives saved is much less clear: evaluations of JSY suggest a massive increase in antenatal care visits and institutional delivery, but the effects on neonatal mortality were modest, with an estimated 2 to 3 deaths per 1000 live births averted ^11,12^..

A primary explanation for the failure of increased uptake of antenatal visits and institutional birth to translate into concomitant gains in survival is poor quality of care ^12,17^. It is estimated that, of 1 million newborn deaths that are amenable to health care worldwide per year, around 60% occurred due to poor quality of care ^18^. Studies have documented gaps in the foundations of care throughout pregnancy and during delivery, including deficits in basic equipment at public primary facilities ^19^, failure to provide essential interventions during antenatal care visits ^20,21^, inadequate referral systems ^22^, and designated delivery facilities without the capacity to provide basic obstetric emergency care ^23^. Observations of providers on the process of care have documented low adherence to clinical guidelines in delivery care ^24^ and lack of attention to essential services such as postnatal care ^25^. Much of the increase in facility delivery under JSY occurred in public clinics ^12^, where quality deficits are the most pronounced ^19,26^. While India’s Newborn Action Plan, enunciated in 2014 to accelerate progress against newborn mortality and stillbirth, emphasizes quality of care, it is a statement of principles that provides only general recommendations on regulation and standards, organizational capacity, and models of care ^27^. How facilities can achieve the Indian Public Health Standards and whether reaching the levels of inputs to care defined in the standards will be sufficient to improve outcomes is not clearly established ^19^.

It is difficult to quantify the level of quality required to translate institutional delivery into increased survival and to define the potential impact of health system quality on newborn survival due to challenges in measuring quality of care and due to overrepresentation of higher risk deliveries in higher quality facilities. Thus, our study was performed with several aims. First, we aimed to characterize health system quality for maternity and newborn care in India at the district level. Since the birth outcomes are shaped by multiple paths in the continuum of care ranging from ante, intra, to postpartum period, we tried to encompass all maternity services in our quality indicator. Second, we aimed to assess if institutional delivery has a positive effect on newborn survival overall and if the effect of institutional delivery on survival depends on the quality of the maternal and newborn health systems. Third, we quantified the potential newborn lives that could be saved by institutional delivery in districts with better health system quality in India.

## Methods

### Data source

Data were taken from the National Family Health Survey (NFHS) IV, a nationally representative household survey conducted in two phases from January 2015 to December 2016 in all 29 states and 7 union territories in India.

### Study design and sample

A stratified two-stage sampling design was adopted for both urban and rural areas. The primary sampling units (PSUs)—villages or census enumeration blocks—were selected with probability proportional to population size. Households were selected by systematic random sampling within each PSU. The overall response rate for the survey was 98%. Further details can be found elsewhere ^28^. We defined the study sample as the most recent birth (singleton or multiples) in the 5 years preceding the survey based on interviews with women aged 15 to 49 years.

### Outcomes

The primary outcome was neonatal mortality: death within the first 28 days of life. In sensitivity analysis, we defined early neonatal mortality as death within 7 days of live birth.

### Maternal health service use and quality

We defined institutional delivery as maternal report of delivering at a formal health facility. We characterize health service quality for maternity and newborn services at the district level. In India, state is the political unit at which federal policies operate and the budgetary allocation for different sectors of development is determined ^29^. Districts are the administrative level below states, and form the lowest administrative unit at which the provision of services and infrastructure is planned and implemented ^30^. With increasing decentralization of health services, the district is an important administrative unit for budgeting, planning, and implementing health programs ^31^. District hospitals act as referral centers for all public facilities within district boundaries. Since distance is an important factor in women’s choice of where to deliver in India, particularly for poorer women ^32,33^, we assume there is minimal travel outside of districts for health services.

To characterize the quality of the maternal and newborn care experienced during pre, intra, and postpartum period in each district, we relied on maternal report of content of antenatal (ANC) and postnatal (PNC) care among women who had used the formal health system for each service. The content of intrapartum care is not typically measured via self-report due to validity concerns ^34,35^.

We reviewed guidelines for ANC and PNC provided by the World Health Organization (WHO) to list essential services to be provided by the health system during pregnancy and the postpartum period ^36,37^ and identified corresponding items in NFHS: 7 items for ANC (being weighed, having blood pressure taken, having urine sample taken, having blood sample taken, being given or bought iron tablets/syrup, receiving tetanus injection, receiving ultrasound testing ^36^) and 4 for PNC (newborn weighed after birth; examined within 1 h after birth; examined by a doctor, nurse or midwife; and put to breast within 1 h).

Women who used formal health care were assigned a score calculated as the proportion of items received per service; these were each averaged by district to capture district-level service quality for ANC and for PNC. The district scores for ANC and PNC were averaged together for the final district-level health system quality score. Districts were grouped into quintiles of lowest to highest quality score (Supplementary Fig. 1).

### Covariates

We applied the conceptual framework of Mosley and Chen ^38^ to consider factors linked to neonatal survival at multiple levels, and we identified key covariates at the child, maternal, household, and district levels following review of prior analyses in India ^39–41^. Child’s gender, multiple birth, and a categorical indicator of birth order and birth interval (first birth, 2nd or 3rd birth with interval up to 24 months, 2nd or 3rd birth with interval over 24 months, 4th or higher birth with interval up to 24 months, 4th or higher birth with interval over 24 months) were included. Maternal characteristics include mother’s age in 5-year groups, marital status, educational attainment, and prior pregnancy ending in miscarriage or stillbirth. Household-level variables include wealth quintile based on the asset index ^28^ and urban vs. rural residence. District economic status was calculated as the proportion of households in the two lowest wealth quintiles per district.

### Statistical analysis

We reported characteristics of the final analytic sample, including crude death rate by each covariate with 95% Confidence interval (CI), and content of care items at the individual level. We produced the maps presenting the distribution of quintiles of district health system quality scores and prevalence of institutional delivery at the district level across the country.

To test the relationships of institutional delivery and district maternal and newborn health system quality with neonatal mortality, we used a three-level random intercept model with newborns nested within district nested within state. A number of factors operate across multiple geographic levels to shape the variation in various health outcomes including neonatal mortality. Therefore, single-level analyses may lead to over- or under-estimation of the effect of factors. Technical details are included in the Supplemental Material. First, we analyzed a null model with no predictor variables. Model 1 included the first exposure of institutional delivery, controlling for individual, maternal, and district characteristics. In model 2 we added the second exposure, district-level maternal and newborn health system quality. Finally, we tested the cross-level interaction between district-level maternal and newborn health system quality score and institutional delivery (Model 3). We calculated the variance partition coefficient (VPC), the proportion of variation in the log odds of neonatal death attributable to each level, and the proportional change in variance (PCV) in the log odds of neonatal death explained by covariates in the model.

To quantify the difference in neonatal mortality between home and institutional delivery as health system quality changes, we repeated model 1 within strata of maternal and newborn health system quality. We predicted prevalence of neonatal mortality for each delivery location by stratum, holding other covariates at their stratum-specific mean.

We assessed the validity of our maternal and newborn health system quality indicator through a falsification test where we replaced the outcome variable of neonatal death with the recent occurrence of diarrhea in children under 5, an outcome that could be affected by confounders of the relationship between maternal and newborn health service quality and neonatal mortality but should not be directly affected by maternal and newborn health service quality. We repeated the main analyses using early neonatal mortality as the outcome.

Sampling weights were used for all analyses; bivariate analysis of neonatal mortality incorporates survey stratification as well. STATA 16.0 (StataCorp, College Station, Texas) was used for data preparation and all multilevel models. Maps were created using ArcMap10.7.1 (ESRI, Redlands, California).

### Ethical approval

All methods were carried out in accordance with the relevant guidelines and regulations. The Demographic Health Surveys Program obtained ethical approval from the Ethics Review Board at the International Institute for Population Sciences, Mumbai before the surveys were implemented, with written informed consent obtained from participants during the survey. No further ethical approval was needed for this secondary analysis of publicly available data.

## Results

Of the 192,671 eligible births, 10 were missing information on the child’s age at death, 605 on birth order or birth interval, and 102 on the place of delivery, which led to 708 cases with at least one missing variable and a final analytic sample of complete cases including 191,963 births across 640 districts within 36 states and union territories (Table 1). The characteristics of excluded observations are provided in Supplementary Table 1. The proportion of neonatal death and twin or triplet was higher in the excluded sample compared to the analytic sample.

**Table 1:** Descriptive statistics of analytic sample (N = 191,963).

	Total	Neonatal mortality
	(N = 191,963)	% (95% CI)
**Proportion district HH in poorest/poor quintiles**		
Mean (SD)	0.45 (0.26)	
**Gender**		
Male	104,498 (54.4%)	1.94 (1.84, 2.05)
Female	87,466 (45.6%)	1.78 (1.67, 1.90)
**Birth order and interval**		
1st	64,697 (33.7%)	1.89 (1.76, 2.03)
2nd or 3rd, interval ≤ 24 months	28,028 (14.6%)	1.90 (1.70, 2.12)
2nd or 3rd, interval > 24 months	69,689 (36.3%)	1.30 (1.19, 1.41)
≥ 4th, interval ≤ 24 months	8,038 (4.2%)	4.47 (3.94, 5.06)
≥ 4th, interval > 24 months	21,514 (11.2%)	2.64 (2.39, 2.91)
**Multiple**		
Singleton	188,999 (98.5%)	1.73 (1.65, 1.80)
Twin or triplet	2,965 (1.5%)	10.97 (9.34, 12.85)
**Maternal age (years)**		
≤ 20	6,505 (3.4%)	3.33 (2.82, 3.93)
21–24	60,002 (31.3%)	1.89 (1.75, 2.03)
25–29	72,150 (37.6%)	1.57 (1.45, 1.70)
30–34	35,280 (18.4%)	1.77 (1.61, 1.94)
35–39	13,337 (6.9%)	2.37 (2.07, 2.71)
40–44	3,629 (1.9%)	3.09 (2.50, 3.81)
45–49	1,065 (0.6%)	5.03 (3.71, 6.78)
**Marital status**		
Never married or previously married	2,606 (1.4%)	1.99 (1.45, 2.72)
Currently married	189,358 (98.6%)	1.87 (1.79, 1.95)
**Maternal education level**		
No education	53,135 (27.7%)	2.72 (2.55, 2.90)
Primary	25,823 (13.5%)	2.36 (2.13, 2.61)
Secondary	90,023 (46.9%)	1.45 (1.35, 1.56)
Higher	22,984 (12.0%)	0.98 (0.83, 1.15)
**Previous pregnancy ending in miscarriage or stillbirth**		
No	177,800 (92.6%)	1.82 (1.74, 1.90)
Yes	14,164 (7.4%)	2.46 (2.16, 2.82)
**Wealth quintile**		
Poorest	44,948 (23.4%)	2.87 (2.68, 3.07)
Poor	40,623 (21.2%)	2.22 (2.06, 2.40)
Middle	38,156 (19.9%)	1.80 (1.62, 1.99)
Richer	36,415 (19.0%)	1.18 (1.03, 1.34)
Richest	31,823 (16.6%)	0.89 (0.76, 1.03)
**Residence**		
Urban	56,988 (29.7%)	1.25 (1.12, 1.39)
Rural	134,976 (70.3%)	2.13 (2.04, 2.23)
**Delivery at institution**		
No	35,722 (18.6%)	2.60 (2.39, 2.82)
Yes	156,242 (81.4%)	1.70 (1.62, 1.79)
**District-level health system quality quintile**		
1st (lowest quality)	52,383 (27.3%)	2.97 (2.80, 3.16)
2nd	25,647 (13.4%)	1.93 (1.76, 2.13)
3rd	39,542 (20.6%)	1.61 (1.45, 1.80)
4th	37,983 (19.8%)	1.30 (1.13, 1.48)
5th (highest quality)	36,411 (19.0%)	1.10 (0.96, 1.26)

Overall, 81.4% of births occurred in a formal institution and 3881 neonates (2.0%) died within 28 days of birth. Crude death rate declined across quintiles of district-level maternal and newborn health system quality from 3.0% in the lowest quality quintile to 1.1% in the highest. Districts with the lowest maternal and newborn health system quality were also the most populous, with 27.3% of births taking place in the 128 districts with the worst maternal and newborn health service quality.

Recommended content of care was reported more commonly for ANC than PNC (Table 2), with nearly all women who sought formal ANC receiving a tetanus injection and 76.7% reporting an ultrasound compared to 1 in 5 children checked within an hour of birth. Overall, women using formal health services received 87% of recommended ANC items and 55% of recommended PNC items. The mean district composite score was 0.71 with a standard deviation (SD) of 0.08.

**Table 2:** Service items for antenatal care (ANC) and postnatal care (PNC) at individual and district levels.

ANC quality indicators (N=130,395)			PNC quality indicators (N=150,134)		
	N	(%)		N	(%)
**Weighed**			**Weighed at birth**		
No	12,464	(9.6)	No	7,285	(4.9)
Yes	117,931	(90.4)	Yes	140,962	(93.9)
**Blood pressure taken**			Missing	1,889	(1.3)
No	13,735	(10.5)	**Put to breast ≤ 1 hour**		
Yes	116,660	(89.5)	No	41,368	(27.6)
**Urine sample taken**			Yes	100,538	(67.0)
No	15,960	(12.2)	Missing	8,230	(5.5)
Yes	114,436	(87.8)	**Check by health professional**		
**Blood sample taken**			No	97,778	(65.1)
No	16,655	(12.8)	Yes	50,617	(33.7)
Yes	113,740	(87.2)	Missing	1,740	(1.2)
**Tetanus injection**			**Postnatal check ≤ 1 hour**		
No	6,781	(5.2)	No	118,475	(78.9)
Yes	122,844	(94.2)	Yes	29,740	(19.8)
Missing	770	(0.6)	Missing	1,920	(1.3)
**Iron given or bought**			**Summary (0-1)**	**Mean**	**SD**
No	21,789	(16.7)	Individual ANC score	0.87	(0.20)
Yes	108,257	(83.0)	District ANC score	0.87	(0.10)
Missing	350	(0.3)	Individual PNC score	0.55	(0.25)
**Ultrasound test taken**			District PNC score	0.55	(0.09)
No	30,379	(23.3)	District composite score	0.71	(0.08)
Yes	100,016	(76.7)			

**Figure 1:**
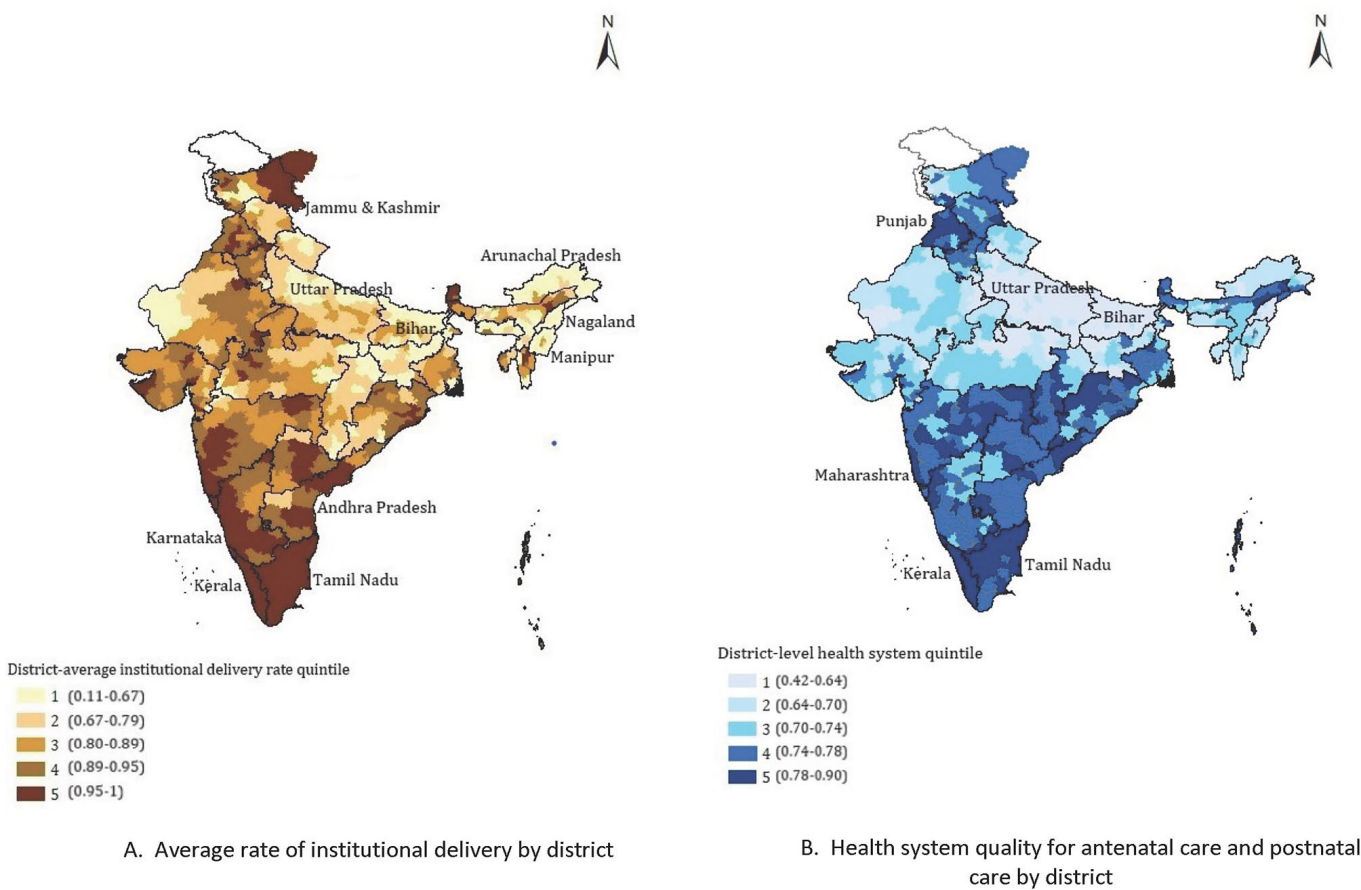
Coverage and quality of maternity and newborn health system in India.

Institutional delivery and institution quality for maternal and newborn care varied across the districts of India (Fig. 1A,B). Institutional delivery and quality were both generally higher in South Indian states such as Tamil Nadu and Kerala. States targeted for national focus due to high poverty and poor health outcomes demonstrated low levels of maternal and newborn health system quality: 36 of 38 districts in Bihar were in the lowest quality quintile, as were most districts in Uttar Pradesh.

Institutional delivery was not significantly associated with neonatal death after adjustment (Table 3, Model 1). District maternal and newborn health service quality score was also not significantly associated with odds of neonatal death overall as shown in Model 2. The interaction between district health system quality score and institutional delivery was statistically significant.

**Table 3:** Association of institutional delivery and district-level health system quality with neonatal mortality (N = 191,963).

Fixed Part		Null	M1			M2			M3		
			AOR	(UCI,	LCI)	AOR	(UCI,	LCI)	AOR	(UCI,	LCI)
Individual-level	Institutional delivery		0.95	(0.80,	1.12)	0.95	(0.80,	1.12)	3.57	(1.47,	8.68)
District-level	District mean score					0.65	(0.16,	2.59)	2.73	(0.38,	19.49)
Cross-level interaction	Institutional delivery × score								0.13	(0.03,	0.53)

Random part		Null	M1			M2			M3		
N*	State	36	36			36			36		
	District	640	640			640			640		
	Individual	191,963	191,963			191,963			191,963		
Variance	State	0.168(0.063)	0.090 (0.032)			0.082 (0.029)			0.074 (0.028)		
	District	0.114(0.046)	0.083(0.042)			0.084(0.042)			0.084(0.042)		
VPC (%)	State	4.5	2.6			2.4			2.1		
	District	3.2	2.4			2.4			2.4		
PCV (%)**	State	–	44.5			49.5			54.5		
	District	–	25.0			24.4			24.0		

M1 –M3: Adjusted for newborn gender, birth order & birth interval, multiple birth, maternal age, marital status, maternal education, previous pregnancy ending in stillbirth or miscarriage, wealth level, urban or rural residence, district-level poverty.

*AOR* Adjusted odds ratio, *VPC* Variance partition coefficient,* PCV* Proportional change in variance.

*N: 10 observations were excluded in null model due to missing values in outcome variable. An additional 698 were excluded in M1 ~ M3 due to missing values in the independent variables (refer to Table 1).

**PCV of M1–M3: calculated compared to null model.

**Figure 2:**
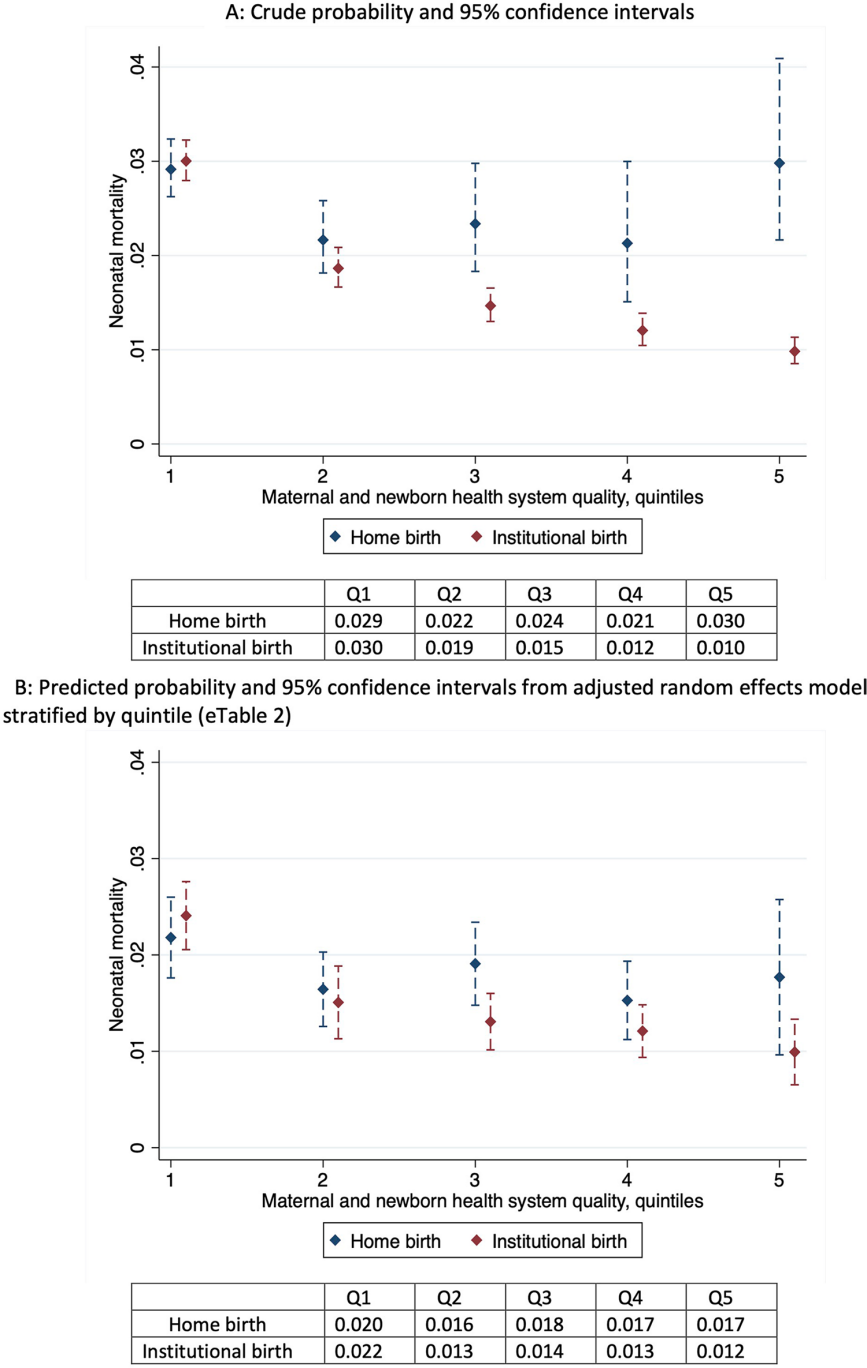
Neonatal mortality by quintile of district-level maternal and newborn health system quality.

Results from the model stratified by quintile are shown in Supplementary Table 2; institutional delivery was associated with reduced odds of mortality in quintiles 3 (AOR 0.68, 95% CI 0.47, 0.98) through 5 (AOR 0.56, 95% CI 0.33, 0.95), but not in the second-lowest quality quintile (AOR 0.92, 95% CI 0.65, 1.29). In the lowest quality quintile districts, the institutional delivery was associated with increased odds of neonatal mortality (AOR 1.11, 95% CI 1.05, 1.17). As shown in Fig. 2A, crude probability of neonatal mortality for institutional deliveries declines across quality quintiles of the maternal and newborn health system, such that in the highest quality quintile, mortality is 10 deaths per 1000 births for institutional deliveries compared to 30 per 1000 for home births. Adjustment for individual and district characteristics narrowed but did not close this gap: predicted mortality was higher among institutional births in districts with the lowest quality quintile, but lower by 8 births per 1000 in the highest quality quintile (18 deaths per 1000 home births compared to 10 deaths per 1000 births in health facilities) holding other covariates at their stratum-specific mean (Fig. 2B).

The portion of the variation in neonatal death attributed to state and district was small relative to variation between individuals within district (VPC: 4.5% and 3.2% for state and district respectively in the null model). Of the variance in neonatal death attributable to each of these levels, 54.5% of between-state differences and 24.0% of between-district differences were explained by the addition of predictor variables, resulting in the reduction in VPC from 4.5 to 2.1% at state level and from 3.2 to 2.4% at district level (Table 3).

Sensitivity analyses supported the main findings. Results were similar for early neonatal death, with a significant interaction between district health system quality and institutional delivery (Supplementary Table 3, Supplementary Fig. 2). The falsification test showed some protective effect of maternal and neonatal health system quality but no significant protective effect on occurrence of recent diarrhea due to institutional delivery or the interaction between maternal and neonatal health system quality and institutional delivery, which argues against unmeasured confounding of these analyses (Supplementary Table 5).

## Discussion

This analysis of 191,963 births in India found that without high-quality district health systems, health system coverage is insufficient to save newborn lives. Institutional delivery was not associated with increased newborn survival in districts with low quality or was even negatively associated with newborn survival in districts with the lowest maternal and newborn health service quality—and the most births—even after accounting for individual characteristics. Current policies and expenditure mostly aim at increasing institutional delivery. However, our findings indicate that they will fail to improve newborn survival where health system quality for maternity services is inadequate, and will in fact worsen inequities in health outcomes, with those in the highest quality districts benefitting the most from improved health system coverage and those in the lowest quality districts not at all. These findings underscore the need to address both access and quality of health services in efforts to reduce the burden of avertable newborn deaths.

The finding of a difference of 8 deaths per 1,000 live births between home and institutional delivery in districts in the highest quintile of quality score adds to a small number of empirical studies on the potential population health impact of access to high-quality maternity care ^42,43^. This analysis further builds on work with the same dataset that classified quality at the district level as high or low based on residual mortality after accounting for institutional delivery and found that district-level quality had a stronger association with neonatal mortality than did the use of ANC and facility delivery ^44^. Analyses of other national surveys in India have documented the importance of receipt of evidence-based interventions such as tetanus injection—a potential proxy for higher quality maternal health systems—in the observed reduction in neonatal mortality at an individual level ^8^. Our work uses a broader measure of quality of care in an effort to test the association of maternal and newborn health system quality with neonatal mortality.

Existing national programs such as JSY have demonstrated tremendous potential in increasing uptake of maternal health services ^11,12,45^. Such policy levers must be combined with a greater focus on health system quality to achieve gains in neonatal mortality moving forward. Given the finding that a low proportion of variability in mortality is explained at the district level, efforts to strengthen health services will need to be paired with interventions to address within-district inequities in health status to yield true population benefit. Pervasive gaps in quality of care and stark inequities between north and south India in this analysis echo findings from the late 1990s onwards ^8,21^, suggesting that policy initiatives from the National Rural Health Mission to JSY have not fully redressed long-standing inequities. Improving service quality at the district level across wide swaths of the country will require an appropriately systems-oriented approach that intervenes in the whole spectrum of pre-pregnancy, pregnancy, delivery, and post-delivery period ^17^. Much existing quality improvement research assesses interventions for individual providers and facilities at the point of care, such as provider training or checklists, that have shown modest and short-term effects ^46^. One of the most rigorous of such studies, the Better Birth Trial, was a randomized controlled trial of an 8-month coaching program to implement a safe childbirth checklist in 120 facilities in Uttar Pradesh. Data from over 150,000 deliveries demonstrated that the intervention improved adherence to clinical guidelines but did not reduce death or severe outcomes in the mother-newborn dyad ^47^. Point of care interventions during labor and delivery, even those that produce improvements in one element of a high-quality health system, are unlikely to yield gains in health outcomes at scale in the absence of a more comprehensive scope of approaches during the whole maternity period and improvement to the underlying health system foundations. Improving quality in districts in poorly performing states like Uttar Pradesh may require new strategies to imbue quality at the core of health service delivery. Such strategies require shifts in the governance of health systems, including much stronger measurement and accountability, service models that provide immediate emergency care to mothers and newborns with complications, financing that supports excellent performance, and a competent and motivated workforce ^17,48^.

The study findings are subject to several limitations. First, we used a proxy for the quality of all maternal and newborn health services in the district based on an assumption that reported receipt of interventions during the antenatal and immediate postnatal periods will be correlated with unmeasured aspects that relate directly to better newborn survival such as timely and competent delivery care. A few existing studies suggest modest correlation among quality of antenatal, intrapartum, and postnatal care in Uttar Pradesh and countries in sub-Saharan Africa ^20,49,50^, but further support for this assertion in this setting is required, particularly given variation in individual experience within districts. Second, our analyses are based on the assumption that the mothers used the health facilities located within the districts they are residing in. However, some pregnant mothers living in the district border or who were referred to higher-level hospitals due to emergencies may have used the services outside their residential district although it is expected to be rare. In considering the influence of health system quality throughout pregnancy, including but not limited to delivery, we focused on the district as the most relevant health system for the majority of women and the relevant level for health service policy given decentralization. Health system quality is heterogeneous within states while care seeking is heterogeneous within community; use of these larger or smaller units would increase potential misclassification error. Third, the quality score is based on maternal self-report of services received up to 5 years before, which may be subject to error; however, recent evidence suggests that the validity of ANC and PNC content indicators is highest for those regarding discrete clinical activities such as the items used here ^51^. Evidence is needed from India on the validity of maternal recall for these items. Fourth, the cross-sectional design prevents the interpretation of the causality. Mothers and newborns in districts with lower maternal and newborn health system quality differ from those in districts with higher quality; the results should not be taken as evidence that improving health system quality alone is sufficient to reduce neonatal mortality to levels expected in districts with higher quality health system. Finally, it is possible that the observed association is a product of uncontrolled confounding such as unmeasured risks among women who deliver at home or better district governance overall, not just for higher quality health services.

This analysis used nationally representative data to define a measure of district health system quality for maternal and newborn services throughout India and to assess the association of institutional delivery with neonatal death in districts with lower and higher quality. We quantified the survival benefit of higher quality health systems and found that institutional delivery does not confer a survival benefit on newborns in settings of low health system quality. These models suggest that the combination of improved district health systems and near-universal institutional delivery may be required to put India on the path to minimizing avertable newborn mortality. As countries seek to expand provision of core services, system-wide improvement in quality needs to be a central part of that effort.

## Supplementary Information

Supplementary Information.

## Data Availability

National Family Health Survey data can be obtained from a public, open access repository through registration (https://dhsprogram.com/). No additional data were used.

## References

[1] Hug L, Alexander M, You D, Alkema L (2019). National, regional, and global levels and trends in neonatal mortality between 1990 and 2017, with scenario-based projections to 2030: A systematic analysis. Lancet Glob. Health.

[2] Mortality rate, neonatal (per 1,000 live births) - India | Data. https://data.worldbank.org/indicator/SH.DYN.NMRT?locations=IN.

[3] Blencowe H (2016). National, regional, and worldwide estimates of stillbirth rates in 2015, with trends from 2000: A systematic analysis. Lancet Glob Health.

[4] Roy MP (2016). Mitigating the stillbirth challenge in India. The Lancet.

[5] Million Death Study Collaborators (2017). Changes in cause-specific neonatal and 1–59-month child mortality in India from 2000 to 2015: A nationally representative survey. Lancet.

[6] Bhutta ZA (2014). Can available interventions end preventable deaths in mothers, newborn babies, and stillbirths, and at what cost?. The Lancet.

[7] Dandona R (2020). Subnational mapping of under-5 and neonatal mortality trends in India: the Global Burden of Disease Study 2000–17. The Lancet.

[8] Singh A, Kumar K, Singh A (2019). What explains the decline in neonatal mortality in India in the last three decades? Evidence from three rounds of NFHS surveys. Stud. Fam. Plann..

[9] Akseer N (2017). Progress in maternal and child health: how has South Asia fared?. BMJ.

[10] Vellakkal S (2017). Has India’s national rural health mission reduced inequities in maternal health services? A pre-post repeated cross-sectional study. Health Policy Plan.

[11] Lim SS (2010). India’s Janani Suraksha Yojana, a conditional cash transfer programme to increase births in health facilities: An impact evaluation. The Lancet.

[12] Powell-Jackson T, Mazumdar S, Mills A (2015). Financial incentives in health: New evidence from India’s Janani Suraksha Yojana. J. Health Econ..

[13] Joe W, Perkins JM, Kumar S, Rajpal S, Subramanian SV (2018). Institutional delivery in India, 2004–14: Unravelling the equity-enhancing contributions of the public sector. Health Policy Plan.

[14] Joshi S, Sivaram A (2014). Does it pay to deliver? An evaluation of India’s safe motherhood program. World Dev..

[15] Randive B, San Sebastian M, De Costa A, Lindholm L (2014). Inequalities in institutional delivery uptake and maternal mortality reduction in the context of cash incentive program, Janani Suraksha Yojana: Results from nine states in India. Soc. Sci. Med..

[16] Lee H-Y, Oh J, Kim R, Subramanian SV (2020). Long-term trend in socioeconomic inequalities and geographic variation in the utilization of antenatal care service in India between 1998 and 2015. Health Serv. Res..

[17] Kruk ME (2018). High-quality health systems in the Sustainable Development Goals era: time for a revolution. Lancet Glob. Health.

[18] Kruk ME (2018). Mortality due to low-quality health systems in the universal health coverage era: a systematic analysis of amenable deaths in 137 countries. The Lancet.

[19] Sharma J, Leslie HH, Regan M, Nambiar D, Kruk ME (2018). Can India’s primary care facilities deliver? A cross-sectional assessment of the Indian public health system’s capacity for basic delivery and newborn services. BMJ Open.

[20] Marchant T (2015). Adding content to contacts: Measurement of high quality contacts for maternal and newborn health in Ethiopia, North East Nigeria, and Uttar Pradesh, India. PLOS ONE.

[21] Rani M, Bonu S, Harvey S (2008). Differentials in the quality of antenatal care in India. Int. J. Qual. Health Care.

[22] Chaturvedi S, Randive B, Diwan V, De Costa A (2014). Quality of obstetric referral services in India’s jsy cash transfer programme for institutional births: A study from Madhya Pradesh Province. PLoS One.

[23] Sabde Y (2016). The availability of emergency obstetric care in the context of the JSY cash transfer programme in Madhya Pradesh, India. BMC Pregn. Childbirth.

[24] Sharma, G. An Investigation into Quality of Care at the Time of Birth at Public and Private Sector Maternity Facilities in Uttar Pradesh, India. (London School of Hygiene & Tropical Medicine, 2017).

[25] Coffey D (2014). Costs and consequences of a cash transfer for hospital births in a rural district of Uttar Pradesh, India. Soc. Sci. Med..

[26] Kaur J, Franzen SRP, Newton-Lewis T, Murphy G (2019). Readiness of public health facilities to provide quality maternal and newborn care across the state of Bihar, India: a cross-sectional study of district hospitals and primary health centres. BMJ Open.

[27] Ministry of Health & Family Welfare. *India Newborn Action Plan (INAP)*. https://nhm.gov.in/index4.php?lang=1&level=0&linkid=153&lid=174 (2014).

[28] International Institute for Population Sciences. *National Family Health Survey (NFHS-4), 2015–2016*. (2017).

[29] Baddeley M, McNay K, Cassen R (2006). Divergence in India: Income differentials at the state level, 1970–97. J. Dev. Studies.

[30] Wanmali S, Islam Y (1995). Rural Services, Rural Infrastructure and Regional Development in India. Geogr. J..

[31] Seshadri SR, Parab S, Kotte S, Latha N, Subbiah K (2016). Decentralization and decision space in the health sector: A case study from Karnataka, India. Health Policy Plan.

[32] Kumar S, Dansereau EA, Murray CJL (2014). Does distance matter for institutional delivery in rural India?. Appl. Econ..

[33] Kesterton AJ, Cleland J, Sloggett A, Ronsmans C (2010). Institutional delivery in rural India: the relative importance of accessibility and economic status. BMC Pregn. Childbirth.

[34] Blanc AK, Diaz C, McCarthy KJ, Berdichevsky K (2016). Measuring progress in maternal and newborn health care in Mexico: validating indicators of health system contact and quality of care. BMC Pregn. Childbirth.

[35] Blanc AK (2016). Assessing the validity of indicators of the quality of maternal and newborn health care in Kenya. J. Glob. Health.

[36] *WHO recommendations on antenatal care for a positive pregnancy experience*. (World Health Organization, 2016). 28079998

[37] World Health Organization (2014). WHO recommendations on postnatal care of the mother and newborn.

[38] Mosley WH, Chen LC (2003). An analytical framework for the study of child survival in developing countries. 1984. Bull World Health Organ.

[39] Bhalotra S, van Soest A (2008). Birth-spacing, fertility and neonatal mortality in India: Dynamics, frailty, and fecundity. J. Economet..

[40] Houweling TAJ (2019). A prediction model for neonatal mortality in low- and middle-income countries: An analysis of data from population surveillance sites in India, Nepal and Bangladesh. Int. J. Epidemiol..

[41] Goudar SS (2015). Institutional deliveries and perinatal and neonatal mortality in Southern and Central India. Reprod. Health.

[42] Leslie HH, Fink G, Nsona H, Kruk ME (2016). Obstetric Facility Quality and Newborn Mortality in Malawi: A Cross-Sectional Study. PLoS Med..

[43] Chou VB, Walker N, Kanyangarara M (2019). Estimating the global impact of poor quality of care on maternal and neonatal outcomes in 81 low- and middle-income countries: A modeling study. PLoS Med..

[44] Kim R, Choi N, Subramanian SV, Oh J (2018). Service Quality beyond access: A multilevel analysis of neonatal, infant, and under-five child mortality using the Indian demographic and health survey 2015–2016. Perspect. Nurs. Sci..

[45] Carvalho N, Rokicki S (2019). The impact of India’s Janani Suraksha Yojana conditional cash transfer programme: A replication study. The J. Dev. Studies.

[46] Rowe AK (2018). Effectiveness of strategies to improve health-care provider practices in low-income and middle-income countries: A systematic review. Lancet Glob. Health.

[47] Semrau KEA (2017). Outcomes of a Coaching-Based WHO safe childbirth checklist program in India. N. Engl. J. Med..

[48] Ahmed SM (2016). Cross-country analysis of strategies for achieving progress towards global goals for women’s and children’s health. Bull. World Health Organ..

[49] Sharma J, Leslie HH, Kundu F, Kruk ME (2017). Poor quality for poor women? Inequities in the quality of antenatal and delivery care in Kenya. PLOS ONE.

[50] Owili PO, Muga MA, Mendez BR, Chen B (2017). Quality of maternity care and its determinants along the continuum in Kenya: A structural equation modeling analysis. PLoS ONE.

[51] McCarthy KJ, Blanc AK, Warren C, Bajracharya A, Bellows B (2020). Validating women’s reports of antenatal and postnatal care received in Bangladesh, Cambodia and Kenya. BMJ Global Health.

