# Supplementary material for: The association between institutional delivery and neonatal mortality based on the quality of maternal and newborn health system in India
Source: Sci Rep. 2022 Apr 13;12:6220. doi: 10.1038/s41598-022-10214-y (PMC9007995; doi:10.1038/s41598-022-10214-y)
Supplement: Supplementary file 1 — Supplementary Information. [file 41598_2022_10214_MOESM1_ESM.docx]

**Model specification**

The model takes the form:

$$\mathrm{logit}\left( \pi_{ijk} \right)=log(\frac{\pi_{ijk}}{1-\pi_{ijk}})= \beta_{0}+{{\beta_{1}X}^{'}}_{ijk}+{\beta_{2}W'}_{jk}+\left( v_{0k}+u_{0jk} \right)$$

$${[v}_{0k}] \sim N(0, \sigma_{v0}^{2})$$

$$[u_{0jk}] \sim N(0, \sigma_{u0}^{2})$$

This model estimates the log odds of $\pi_{ijk}$ (neonatal or early neonatal death) while adjusting for a vector (${X^{'}}_{\mathrm{ijk}}$) and (${W'}_{jk})$ of independent variables measured at the individual- and district- level respectively. Random effects inside the bracket are residual differentials specific for state *k* ($v_{0k})$ and district *j (*$u_{0jk})$*.* Under the independently and identically distributed assumption, each set of residuals follows a normal distribution.


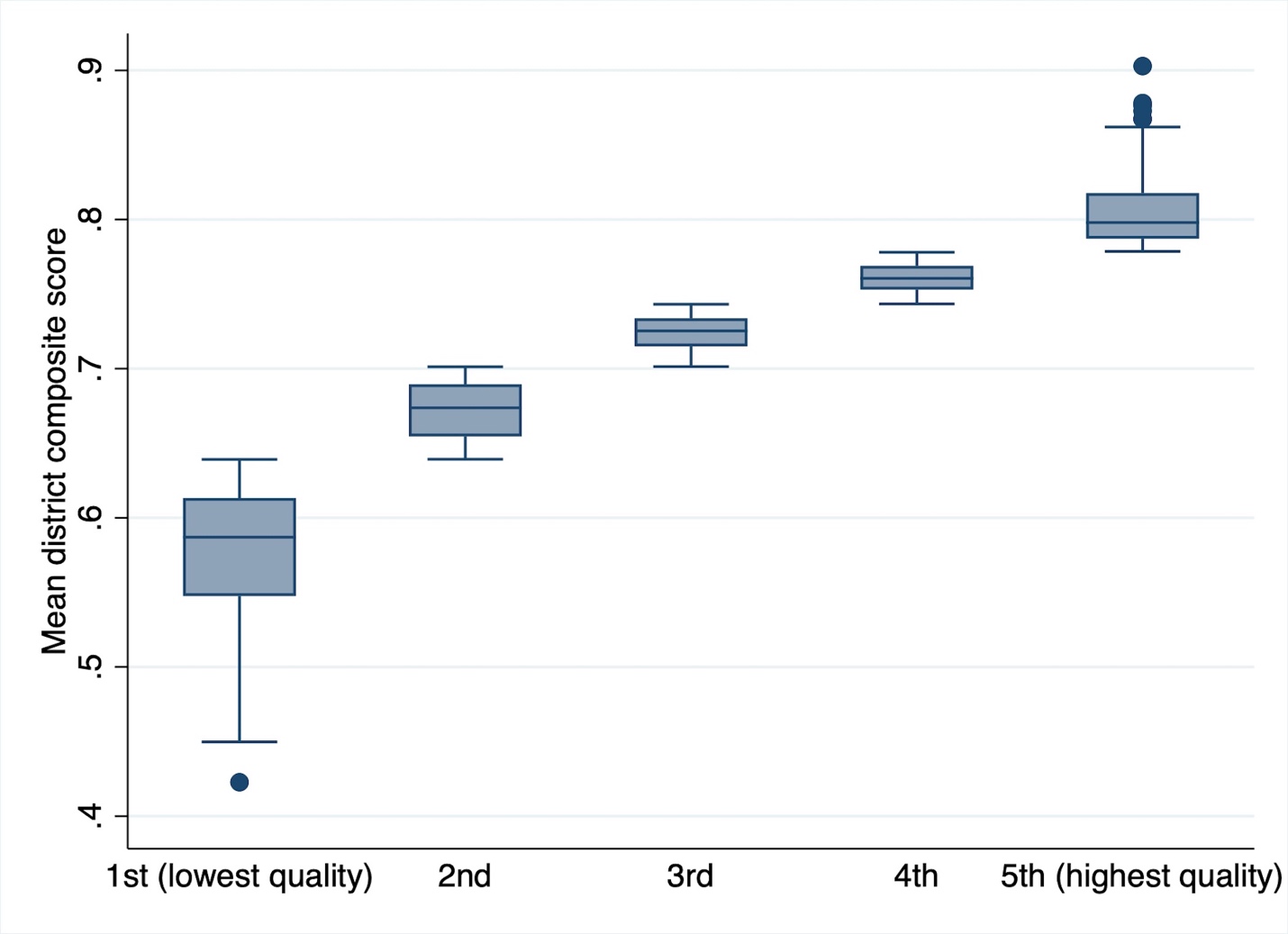


eFigure 1. Distribution of quality score for maternity and newborn health system by quintile

| eTable 1. Respondent characteristics, complete and incomplete cases (unweighted)   \|  \| Incomplete case \| Complete case \| \| --- \| --- \| --- \| \|  \| (N = 708) \| (N = 191,963) \| |
| --- | --- | --- | --- | --- | --- | --- |
| \| **Proportion district HH in poorest/poor quintiles** \|  \|  \| \| --- \| --- \| --- \| \| Mean (SD) \| 0.41 (0.25) \| 0.47 (0.25) \| \| **Neonatal death** \|  \|  \| \| No \| 572 (81.9%) \| 188,208 (98.0%) \| \| Yes \| 126 (18.1%) \| 3,755 (2.0%) \| \| **Gender** \|  \|  \| \| Male \| 353 (49.9%) \| 104,112 (54.2%) \| \| Female \| 355 (50.1%) \| 87,851 (45.8%) \| \| **Birth order and interval** \|  \|  \| \| 1st \| 4 (3.9%) \| 62,399 (32.5%) \| \| 2nd or 3rd, interval ≤ 24 months \| 2 (1.9%) \| 26,712 (13.9%) \| \| 2nd or 3rd, interval > 24 months \| 38 (36.9%) \| 69,031 (36.0%) \| \| ≥ 4th, interval ≤ 24 months \| 4 (3.9%) \| 8,954 (4.7%) \| \| ≥ 4th, interval > 24 months \| 55 (53.4%) \| 24,867 (13.0%) \| \| **Multiple** \|  \|  \| \| Singleton \| 101 (14.3%) \| 189,042 (98.5%) \| \| Twin or triplet \| 607 (85.7%) \| 2,921 (1.5%) \| \| **Maternal age (years)** \|  \|  \| \| ≤20 \| 27 (3.8%) \| 5,906 (3.1%) \| \| 21-24 \| 225 (31.8%) \| 56,341 (29.3%) \| \| 25-29 \| 238 (33.6%) \| 70,602 (36.8%) \| \| 30-34 \| 113 (16.0%) \| 37,650 (19.6%) \| \| 35-39 \| 48 (6.8%) \| 15,507 (8.1%) \| \| 40-44 \| 29 (4.1%) \| 4,592 (2.4%) \| \| 45-49 \| 28 (4.0%) \| 1,365 (0.7%) \| \| **Marital status** \|  \|  \| \| Never married or previously married \| 23 (3.2%) \| 3,268 (1.7%) \| \| Currently married \| 685 (96.8%) \| 188,695 (98.3%) \| \| **Maternal education level** \|  \|  \| \| No education \| 120 (16.9%) \| 55,577 (29.0%) \| \| Primary \| 70 (9.9%) \| 26,845 (14.0%) \| \| Secondary \| 363 (51.3%) \| 89,314 (46.5%) \| \| Higher \| 155 (21.9%) \| 20,227 (10.5%) \| \| **Previous pregnancy ending in miscarriage or stillbirth** \|  \|  \| \| No \| 666 (94.1%) \| 177,479 (92.5%) \| \| Yes \| 42 (5.9%) \| 14,484 (7.5%) \| \| **Wealth quintile** \|  \|  \| \| Poorest \| 106 (15.0%) \| 47,114 (24.5%) \| \| Poor \| 114 (16.1%) \| 43,973 (22.9%) \| \| Middle \| 141 (19.9%) \| 38,595 (20.1%) \| \| Richer \| 157 (22.2%) \| 33,370 (17.4%) \| \| Richest \| 190 (26.8%) \| 28,911 (15.1%) \| \| **Residence** \|  \|  \| \| Urban \| 241 (34.0%) \| 48,087 (25.1%) \| \| Rural \| 467 (66.0%) \| 143,876 (74.9%) \| \| **Delivery at institution** \|  \|  \| \| No \| 29 (4.8%) \| 42,406 (22.1%) \| \| Yes \| 577 (95.2%) \| 149,557 (77.9%) \| \| **District-level health system quality quintile** \|  \|  \| \| 1st (lowest quality) \| 112 (15.8%) \| 49,414 (25.7%) \| \| 2nd \| 126 (17.8%) \| 43,403 (22.6%) \| \| 3rd \| 162 (22.9%) \| 40,333 (21.0%) \| \| 4th \| 158 (22.3%) \| 30,327 (15.8%) \| \| 5th (highest quality) \| 150 (21.2%) \| 28,486 (14.8%) \| |

eTable 2. Stratified analyses of the association between neonatal mortality and institutional delivery by the maternal and newborn care score quintile

|  | | Q1 | | | | Q 2 | | | | Q 3 | | | | Q 4 | | | | Q 5 | | | | |
| --- | --- | --- | --- | --- | --- | --- | --- | --- | --- | --- | --- | --- | --- | --- | --- | --- | --- | --- | --- | --- | --- | --- |
|  | | OR | (UCI, | | LCI) | OR | (UCI, | | LCI) | OR | (UCI, | | LCI) | OR | (UCI, | | LCI) | OR | (UCI, | | LCI) | |
| **Unadjusted** | |  |  | |  |  |  | |  |  |  | |  |  |  | |  |  |  | | |  |
| Fixed Part: Institutional delivery | | 1.05 | (1.00, | | 1.10) | 0.85 | (0.61, | | 1.18) | 0.61 | (0.43, | | 0.86) | 0.62 | (0.45, | | 0.87) | 0.45 | (0.28, | | | 0.73) |
|  | | AOR | (UCI, | | LCI) | AOR | (UCI, | | LCI) | AOR | (UCI, | | LCI) | AOR | (UCI, | | LCI) | AOR | (UCI, | | | LCI) |
| **Fully adjusted^1)^** | |  |  | |  |  |  | |  |  |  | |  |  |  | |  |  |  | | |  |
| Fixed Part: Institutional delivery | | 1.11 | (1.05, | | 1.17) | 0.92 | (0.65, | | 1.29) | 0.68 | (0.47, | | 0.98) | 0.79 | (0.60, | | 1.04) | 0.56 | (0.33, | | | 0.95) |
| Random part^2)^ | | | | | | | | | | | | | | | | | | | | | | |
| N | State | 11 | | | | 19 | | | | 24 | | | |  | 23 | |  |  | 20 | |  | |
|  | District | 128 | | | | 128 | | | | 128 | | | |  | 128 | |  |  | 128 | |  | |
|  | Individual | 49,414 | | | | 43,403 | | | | 40,333 | | | | 30,327 | | | | 28,486 | | | | |
| Variance | State | 0.034 | | (0.017) | | 0.058 | | (0.027) | | 0.065 | | (0.030) | | 0.059 | | (0.038) | | 0.133 | | (0.076) | | |
|  | District | 0.030 | | (0.006) | | 0.019 | | (0.018) | | 0.055 | | (0.051) | | 0.284 | | (0.125) | | 0.213 | | (0.103) | | |
| VPC (%)^3)^ | State | 1.0 | | | | 1.7 | | | | 1.9 | | | | 1.6 | | | | 3.4 | | | | |
|  | District | 0.9 | | | | 0.6 | | | | 1.6 | | | | 7.8 | | | | 5.9 | | | | |
| MOR^3)^ | State | 1.19 | | | | 1.26 | | | | 1.28 | | | | 1.26 | | | | 1.40 | | | | |
|  | District | 1.18 | | | | 1.14 | | | | 1.25 | | | | 1.66 | | | | 1.55 | | | | |

1. Adjusted for newborn gender, birth order & birth interval, multiple birth, maternal age, marital status, maternal education, previous pregnancy ending in stillbirth or miscarriage, wealth level, urban or rural residence, district-level poverty.
2. From fully adjusted model.
3. AOR: Adjusted odds ratio, VPC: Variance partition coefficient, MOR: Median odds ratio.

eTable 3. Association of institutional delivery and district-level maternal and newborn care quality with early neonatal mortality within 7 days

|  | | Null | M1^1)^ | | | M2^1)^ | | | M3^1)^ | | | | |
| --- | --- | --- | --- | --- | --- | --- | --- | --- | --- | --- | --- | --- | --- |
| Response | |  | AOR | (UCI, | LCI) | AOR | (UCI, | LCI) | AOR | | (UCI, | | LCI) |
| Fixed Part | |  |  |  |  |  |  |  |  | |  | |  |
| Individual level | Institutional delivery |  | 0.99 | (0.82, | 1.19) | 0.99 | (0.82, | 1.19) | 3.87 | | (1.53 | | 9.82) |
| District-level | District mean score |  |  |  |  | 0.48 | (0.12, | 1.95) | 2.17 | | (0.26, | | 17.85) |
| Cross-level interaction | Institutional delivery x score |  |  |  |  |  |  |  | 0.12 | | (0.03, | | 0.56) |
|  | Random part |  |  |  |  |  |  |  |  | |  | |  |
| N^2)^ | State | 36 | 36 | | | 36 | | |  | 36 | |  | |
|  | District | 640 | 640 | | | 640 | | |  | 640 | |  | |
|  | Individual | 191,963 | 191,963 | | | 191,963 | | | 191,963 | | | | |
| Variance | State | 0.162(0.060) | 0.073 (0.026) | | | 0.060(0.023) | | | 0.054(0.022) | | | | |
|  | District | 0.132(0.054) | 0.094(0.049) | | | 0.095(0.049) | | | 0.095(0.048) | | | | |
| VPC (%)^3)^ | State | 4.5 | 2.1 | | | 1.8 | | | 1.6 | | | | |
|  | District | 3.7 | 2.7 | | | 2.7 | | | 2.8 | | | | |
| PCV (%)^3),4)^ | State | - | 53.1 | | | 61.2 | | | 65.0 | | | | |
|  | District | - | 26.5 | | | 25.6 | | | 25.5 | | | | |
| MOR^3)^ | State | 1.47 | 1.29 | | | 1.26 | | | 1.25 | | | | |
|  | District | 1.41 | 1.34 | | | 1.34 | | | 1.34 | | | | |

1. M1 ~ M3: Adjusted for newborn gender, birth order & birth interval, multiple birth, maternal age, marital status, maternal education, previous pregnancy ending in stillbirth or miscarriage, wealth level, urban or rural residence, district-level poverty.
2. N: 10 observations were excluded in null model due to missing values in outcome variable. Additional 698 were excluded in M1~M3 due to missing values in the independent variables (refer to Table 1)
3. AOR: Adjusted odds ratio, VPC: Variance partition coefficient, PCV: Proportional change in variance, MOR: Median odds ratio.
4. PCV of M1~M3: calculated compared to null model

eTable 4. Stratified analyses of the association between early neonatal mortality within 7 days after birth and institutional delivery by the maternal and newborn care score quintile

|  | | Q1 | | | | Q 2 | | | | Q 3 | | | | Q 4 | | | | Q 5 | | | |
| --- | --- | --- | --- | --- | --- | --- | --- | --- | --- | --- | --- | --- | --- | --- | --- | --- | --- | --- | --- | --- | --- |
|  | | OR | (UCI, | | LCI) | OR | (UCI, | | LCI) | OR | (UCI, | | LCI) | OR | (UCI, | | LCI) | OR | (UCI, | | LCI) |
| **Unadjusted** | |  |  | |  |  |  | |  |  |  | |  |  |  | |  |  |  | |  |
| Fixed Part: Institutional delivery | | 1.11 | (1.09, | | 1.13) | 0.82 | (0.60, | | 1.11) | 0.70 | (0.50, | | 0.97) | 0.67 | (0.44, | | 1.01) | 0.40 | (0.22, | | 0.73) |
|  | | AOR | (UCI, | | LCI) | AOR | (UCI, | | LCI) | AOR | (UCI, | | LCI) | AOR | (UCI, | | LCI) | AOR | (UCI, | | LCI) |
| **Fully adjusted**^1)^ | |  |  | |  |  |  | |  |  |  | |  |  |  | |  |  |  | |  |
| Fixed Part: Institutional delivery | | 1.20 | (1.18, | | 1.23) | 0.88 | (0.65, | | 1.21) | 0.80 | (0.57, | | 1.13) | 0.88 | (0.62, | | 1.24) | 0.48 | (0.26, | | 0.90) |
| Random part^2)^ | |  |  | |  |  |  | |  |  |  | |  |  |  | |  |  |  | |  |
| N | State | 11 | | | | 19 | | | | 24 | | | | 23 | | | | 23 | | | |
|  | District | 128 | | | | 128 | | | | 128 | | | | 128 | | | | 128 | | | |
|  | Individual | 49,414 | | | | 43,403 | | | | 40,333 | | | | 30,327 | | | | 28,486 | | | |
| Variance | State | 0.031 | | (0.014) | | 0.068 | | (0.055) | | 0.093 | | (0.048) | | 0.047 | | (0.031) | | 0.055 | | (0.077) | |
|  | District | 0.022 | | (0.009) | | 0.036 | | (0.036) | | 0.074 | | (0.066) | | 0.316 | | (0.144) | | 0.264 | | (0.143) | |
| VPC (%)^3)^ | State | 0.9 | | | | 2.0 | | | | 2.7 | | | | 1.3 | | | | 1.5 | | | |
|  | District | 0.7 | | | | 1.1 | | | | 2.2 | | | | 8.7 | | | | 7.3 | | | |
| MOR^3)^ | State | 1.18 | | | | 1.28 | | | | 1.34 | | | | 1.23 | | | | 1.25 | | | |
|  | District | 1.15 | | | | 1.20 | | | | 1.30 | | | | 1.71 | | | | 1.63 | | | |

1. Adjusted for newborn gender, birth order & birth interval, multiple birth, maternal age, marital status, maternal education, previous pregnancy ending in stillbirth or miscarriage, wealth level, urban or rural residence, district-level poverty.
2. From fully adjusted model.
3. AOR: adjusted odds ratio, VPC: Variance partition coefficient, MOR: Median odds ratio.

| 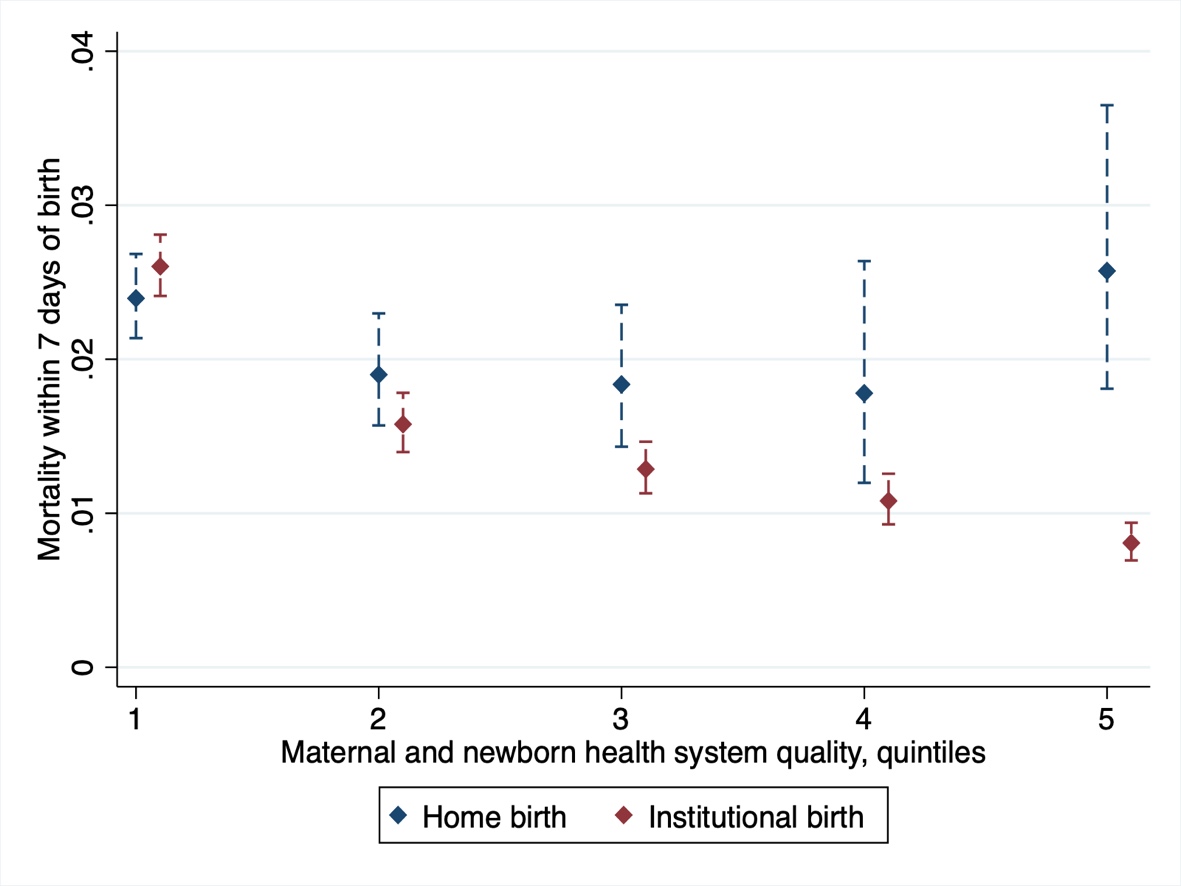   \|  \| Q1 \| Q2 \| Q3 \| Q4 \| Q5 \| \| --- \| --- \| --- \| --- \| --- \| --- \| \| Home birth \| 0.024 \| 0.019 \| 0.018 \| 0.018 \| 0.026 \| \| Institutional birth \| 0.026 \| 0.016 \| 0.013 \| 0.011 \| 0.008 \|   eFigure 2A. Crude probability of early neonatal death by quintile of district-level maternal and newborn care quality |
| --- | --- | --- | --- | --- | --- | --- | --- | --- | --- | --- | --- | --- | --- | --- | --- | --- | --- | --- |
| 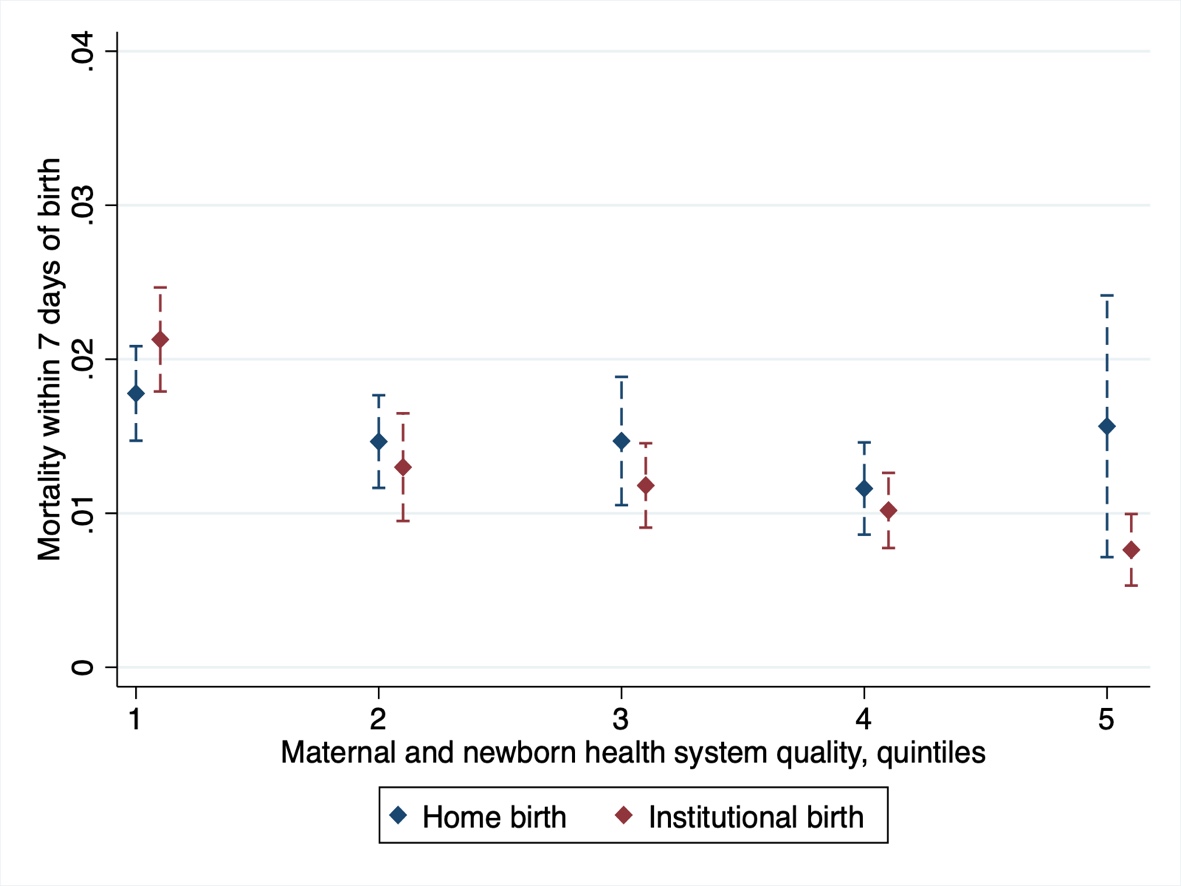   \|  \| Q1 \| Q2 \| Q3 \| Q4 \| Q5 \| \| --- \| --- \| --- \| --- \| --- \| --- \| \| Home birth \| 0.018 \| 0.015 \| 0.015 \| 0.012 \| 0.016 \| \| Institutional birth \| 0.021 \| 0.013 \| 0.012 \| 0.010 \| 0.008 \|   eFigure 2B. Predicted probability of early neonatal death by quintile of district-level maternal and newborn care quality from adjusted random effects model. |

eTable 5. Association of institutional delivery and district-level health system quality score with prevalence of diarrhea in children under 5 (N=185,823)

|  | Institutional delivery | | | District mean score | | | Institutional delivery x score | | |
| --- | --- | --- | --- | --- | --- | --- | --- | --- | --- |
| Outcome: incidence of recent diarrhea | OR | (UCI, | LCI) | OR | (UCI, | LCI) | OR | (UCI, | LCI) |
| M2 | 1.09 | (1.05, | 1.14) | 0.29 | (0.10, | 0.82) |  |  |  |
| M3 | 0.96 | (0.72, | 1.30) | 0.25 | (0.08, | 0.80) | 1.21 | (0.73, | 2.01) |
